# Identifying Unique Versus Shared Pre- and Perinatal Risk Factors for ASD and ADHD Using a Simplex-Multiplex Stratification

**DOI:** 10.1007/s10802-015-0081-0

**Published:** 2015-10-14

**Authors:** Anoek M. Oerlemans, Marlot J. Burmanje, Barbara Franke, Jan K. Buitelaar, Catharina A. Hartman, Nanda N. J. Rommelse

**Affiliations:** Department of Cognitive Neuroscience, Donders Institute for Brain, Cognition and Behavior, Radboud university medical center, Nijmegen, The Netherlands; Karakter, Child and Adolescent Psychiatry University Centre, Reinier Postlaan 12, 6525 GC Nijmegen, The Netherlands; Department of Human Genetics, Radboud university medical center, Nijmegen, The Netherlands; University of Groningen, University Medical Center Groningen, Groningen, Netherlands; Department of Psychiatry, Donders Institute for Brain, Cognition and Behavior, Radboud university medical center, Nijmegen, The Netherlands

**Keywords:** Autism Spectrum Disorders (ASD), Attention-Deficit/Hyperactivity Disorder (ADHD), Simplex-multiplex stratification, Prenatal and perinatal risk factors

## Abstract

Autism spectrum disorder (ASD) and attention-deficit/hyperactivity disorder (ADHD) frequently co-occur. Besides shared genetic factors, pre- and perinatal risk factors (PPFs) may determine if ASD, ADHD, or the combination of both disorders becomes manifest. This study aimed to test shared and unique involvement of PPFs for ASD and ADHD, using an approach that stratifies the sample into affected/unaffected offspring and single-incidence (SPX) versus multi-incidence (MPX) families. Pre- perinatal data based on retrospective parent-report were collected in 288 children (71 % males) from 31 SPX and 59 MPX ASD families, 476 children (65 % males) from 31 SPX and 171 MPX ADHD families, and 408 control children (42 % males). Except for large family size and more firstborns amongst affected offspring, no shared PFFs were identified for ASD and ADHD. PPFs predominantly related to ASD (maternal infections and suboptimal condition at birth) were more often reported in affected than unaffected siblings. PPFs associated with ADHD (low parental age, maternal diseases, smoking and stress) were shared between affected and unaffected siblings. Firstborn-ship was more frequent in SPX than MPX ASD probands. Our results suggest that the co-morbidity of ASD and ADHD is not likely explained by shared PPFs. Instead, PPFs might play a crucial role in the developmental pathways leading up to either disorder. PPFs in ADHD appear to index an increased shared risk, whereas in ASD PPFs possibly have a more determining role in the disorder. SPX-MPX stratification detected possible etiological differences in ASD families, but provided no deeper insight in the role of PPFs in ADHD.

**Electronic supplementary material:**

The online version of this article (doi:10.1007/s10802-015-0081-0) contains supplementary material, which is available to authorized users.

Autism spectrum disorder (ASD) and attention deficit/hyperactivity disorder (ADHD) are both highly heritable, impairing neurodevelopmental disorders that manifest early in development and frequently co-occur (Lichtenstein et al. 2010). ASD is characterized by impairments in social interaction, deficits in verbal and non-verbal communication and by restricted or repetitive patterns of behavior and interests. ADHD is characterized by symptoms of hyperactivity, impulsivity and/or inattention (American Psychiatric Association 2013). High co-morbidity might be explained by shared genetic factors, as indicated by twin studies (Ronald et al. 2008). However, genetic effects do not account for all phenotypic covariation (Ronald et al. 2008), implying that the high co-morbidity rates of ASD and ADHD might also be explained by other factors, such as shared pre- and perinatal risk factors (PPFs) (Rutter and Silberg 2002).

PPFs have proven important in the etiology of both ASD and ADHD. However, since ASD or ADHD have been studied mostly in isolation, we have little knowledge about whether PPFs are shared between the disorders. In meta-analyses of ASD, advanced parental age at birth, maternal prenatal medication use, gestational bleeding, diabetes, being firstborn, fetal distress, birth injury or trauma, low 5-min APGAR score and low birth weight (<5.5 lb or 2500 g) were more frequently observed in individuals with ASD than in controls (Gardener et al. 2009, 2011). Maternal infections, maternal stress, suboptimal condition of the child at birth, prematurity, smoking during pregnancy (Visser et al. 2013), and a birth weight more than two standard deviations above average for gestational age (Abel et al. 2013) were also found related to ASD. Research on ADHD indicates that prenatal exposure to nicotine, alcohol, drugs or toxins, and maternal stress, low birth weight, low maternal age and poor maternal diet are associated with an increased likelihood of developing ADHD (Mill and Petronis 2008; Thapar et al. 2013). Furthermore, an association has been reported between neonatal complications and the severity of ADHD symptoms (Ben Amor et al. 2005). Only two studies so far investigated which early childhood indicators (including PPFs) might be shared between ASD and ADHD, by examining these risk factors in the general population (Jaspers et al. 2013; St St Pourcain et al. 2011). St Pourcain et al. found that maternal smoking might be common to ASD- and ADHD-like symptoms. Jaspers and colleagues reported that male gender and low educational level of the mother were overlapping indicators, yet PPFs such as maternal smoking and low birth weight were specific for ADHD and ASD traits, respectively. All considered, these findings on population-based samples suggest limited overlap of PPFs in ASD and ADHD, except perhaps for low birth weight and maternal smoking. The role of shared PPFs might, however, be different in clinical samples, given greater severity of clinical symptoms among referred cases. In the current study that combines two clinical ASD and ADHD cohorts, we aimed to explore this issue further.

Which role PPFs play in a disorder can be understood by looking at the prevalence of these risk factors in unaffected siblings of patients. The advantage of an affected-unaffected sibling design is that it controls for familial (genetic background and shared environmental) risk factors (Ben Amor et al. 2005). When certain PPFs are present in both affected and unaffected siblings, these factors are probably related to an overall increased risk of developing the disorder (trait factors), without a unique, determining contribution to the disorder. Vice versa, PPFs (predominantly) found in affected offspring but not in unaffected offspring may have a more penetrant, possibly uniquely determining effect on the development of the disorder (state factors). Research comparing affected and unaffected ASD siblings showed that medication use during pregnancy, being firstborn, higher non-optimality scores, low birth weight and low APGAR scores are all relatively more prevalent in autistic children compared to their unaffected siblings (Deykin and MacMahon 1980), suggesting that these factors may explain why the probands did develop ASD, yet their siblings did not. In ADHD families, affected children appear to have significantly higher rates of low birth weight and medical conditions when compared to unaffected siblings, but intriguingly, smoking and alcohol consumption during pregnancy do not appear to differ among siblings (Ben Amor et al. 2005). Recent studies using genetically informative designs suggest the latter factors to be a proxy of ADHD risk genes passed from mother to offspring, rather than predominantly environmental toxic factors (Thapar et al. 2013). The above described research indicates that there might indeed be specific PPFs that are present in affected individuals, but also PPFs that are shared by affected and non-affected (ADHD) siblings. This is the first study to examine which PPFs are related to ASD and/or ADHD and to what extend these factors are uniquely present in affected offspring or shared between affected and unaffected siblings.

A second and more in depth approach that can improve our understanding of the role of PPFs in ASD and ADHD is to stratify families into simplex (SPX) or multiplex (MPX) affected ones. Families with only one affected individual are defined as SPX and families in which two or more individuals are affected, are defined as MPX. The hypothesis is that the constellation of etiological factors differs between SPX versus MPX families. It is expected that individuals from SPX families are more likely than individuals from MPX families to develop the disorder as a result of sporadic genetic (e.g., de novo mutations) or non-genetic causes (e.g., rare PPFs with high penetrance) strictly personal to the patient. These factors may (at least partly) explain why that child developed ASD or ADHD, yet his/her siblings did not. In families with multiple cases of the disorder (MPX families) risk factors may be shared between affected individuals. Previous molecular genetic studies confirmed the assumption of different etiologies for SPX and MPX cases, demonstrating that a more than threefold rate of de novo mutations was identified in ASD SPX families (~7–10 %), compared to ASD MPX families (~2–3 %) or control families (~1 %) (Sebat et al. 2007), indicative of an increased incidence of non-heritable, non-shared risk factors in SPX families. In contrast, it was found that members of MPX families were often in fact less-affected family members, more often exhibiting disorder-related behavioral symptoms and cognitive deficits compared to members of SPX families, indicative of a more pronounced role of shared genetic predispositions (Gerdts et al. 2013; Oerlemans et al. 2015a; Oerlemans et al. 2015b; Virkud et al. 2009). No studies so far have investigated whether differences exist between SPX and MPX ASD and ADHD families. It may be expected that in SPX families, rare, non-shared PPFs with high penetrance that are unique to the affected child and that are not present during the pregnancy and birth of the unaffected siblings are more frequent, whereas common, low penetrance PPFs that increase the overall liability for a disorder are expected to be more frequent in MPX families.

In sum, this is the first study that tests if PPFs for ASD and ADHD are unique or shared using an approach that stratifies the sample into affected versus unaffected offspring and SPX versus MPX-affected families. First, we tested which PPFs were associated with ASD, ADHD or both. Second, we tested whether PPFs only present in affected –but not unaffected- offspring may have a unique, highly penetrant contribution to the disorder (state factors) instead of increasing the overall liability for the disorder only slightly (trait factors). Third, we examined whether offspring from MPX families shares a large proportion of PPFs, whereas in SPX families PPFs were mostly non-shared between affected and unaffected siblings.

## Methods

ASD and ADHD families were recruited as part of two large family-genetic studies: the Biological Origins of Autism (BOA) study and the Dutch part of the International Multicenter ADHD Genetics (IMAGE) study (as described previously in Oerlemans et al. 2015b). Case families were recruited through child psychiatric and pediatric clinics and the Dutch Autism Association (NVA). Families potentially satisfying inclusion criteria received an information brochure and, if interested, were asked to return a pre-stamped response card. Control families were recruited from the same geographical regions as the participating case families via schools and information leaflets. Inclusion criteria for all participants were at least two biological siblings (in case families: at least one child with a clinical diagnosis of ASD or ADHD) and one biological parent willing to participate, European Caucasian descent, offspring age between 4 and 20 years, and offspring IQ ≥ 70. Children with a diagnosis of epilepsy, brain disorders or known genetic disorders, such as Down-syndrome or Fragile-X-syndrome were excluded from participation in order to reduce etiological heterogeneity and provide ASD and ADHD samples with greater clinical homogeneity.

All children and parents were carefully phenotyped for ASD and ADHD using validated and standardized questionnaires and diagnostic interviews, including the Autism Spectrum Quotient (Baron-Cohen et al. 2001), Conners Rating Scales (Conners 1996; Conners et al. 1998), Autism Diagnostic Interview-Revised (Le Couteur et al. 2003), and Parental Account of Childhood Symptoms ADHD subversion (Taylor et al. 1991). Families were then stratified into SPX and MPX based on the number of affected individuals. SPX families were required to have a single-affected proband, a minimum of one male sibling and all siblings and parents of the proband unaffected by ASD or ADHD; MPX families were required to have two or more affected individuals. SPX-MPX stratification was made on the basis of the primary clinical diagnosis of the probands. That is, SPX and MPX ASD families originate from an ASD cohort in which the primary clinical diagnosis of the proband is ASD, SPX and MPX ADHD families originate from an ADHD cohort in which the primary clinical diagnosis of the proband is ADHD.

A total of 288 children from ASD families (including: 56 SPX probands, 96 MPX probands, 81 SPX unaffected siblings, and 55 MPX unaffected siblings), 476 children from ADHD families (including: 31 SPX probands, 270 MPX probands, 47 SPX unaffected siblings, and 128 MPX unaffected siblings), and 408 control children were included in this study, see Table 1 for sample characteristics. For a full description of phenotyping and family classification, see Oerlemans et al. (2015b) or Supplemental Table 1.

**Table 1:** Sample characteristics

	Control families (c)^a^ (*N* = 203)	ASD cohort				ADHD cohort				Group contrasts ASD vs. controls	Group contrasts ADHD vs. controls
		SPX families (*N* = 56)		MPX families (*N* = 59)		SPX families (*N* = 31)		MPX families (*N* = 171)			
		1. ASD probands	2. ASD Unaffected siblings	3. ASD probands	4. ASD Unaffected siblings	5. ADHD probands	6. ADHD Unaffected siblings	7. ADHD probands	8. ADHD Unaffected siblings		
	M (sd)	M (sd)	M (sd)	M (sd)	M (sd)	M (sd)	M (sd)	M (sd)	M (sd)		
Child data											
Number of kids	*N* = 408	*N* = 56	*N* = 81	*N* = 96	*N* = 55	*N* = 31	*N* = 47	*N* = 270	*N* = 128		
Mean family size^b^	2.3	2.7		2.8		2.6		2.5		SPX = MPX > controls	SPX = MPX > controls
Age	11.2 (3.5)	12.0 (3.7)	12.1 (3.8)	11.3 (3.6)	10.9 (4.2)	11.8 (2.4)	10.9 (3.4)	11.4 (2.7)	11.4 (3.6)	1 = 2 = 3 = 4 = c	5 = 6 = 7 = 8 = c
Sex (% males)	41.9	85.7	74.1	72.9	45.5	87.1	74.5	73.6	39.1	1 = 2 = 3 > 4 = c	5 = 6 = 7 > 8 = c
Diagnose (%)											
ASD	0	100	0	100	0	25.8	2.1	14.1	1.6		
ADHD	0	35.7	12.3	43.8	18.1	100	0	100	0		

*Note ASD* autism spectrum disorders; *ADHD* attention-deficit/hyperactivity disorder: *SPX* simplex family; *MPX* multiplex family; *c* controls

^a^control families were combined from both datasets

^b^mean number of children per family

### Pre-and Perinatal Information

Pre- and perinatal information was retrospectively obtained through a questionnaire filled out by the parents before diagnostic assessments. The questionnaire is derived from the Prechtl optimality scales used by Gillberg and Gillberg (1983), with addition of relevant items like parental age, intoxications and maternal stress. The items were grouped into PPFs based on related content following the procedures used successfully in other studies (e.g., Visser et al. 2013), see Table 2. For each factor, a dichotomous variable was created, coding 1 if the risk factor was present and 0 indicating absence of the risk factor. The factor pregnancy after fertility treatment was added, because children born following assisted reproductive technologies (ARTs) have been found to be at higher risk of autism than the general population (Zhan et al. 2013). Further, given that both low and high birth weight and parental age were found to be associated with adverse outcomes, it was decided to examine the effect of these categories separately (Abel et al. 2013).

**Table 2:** Pre-and perinatal risk factors

Prenatal	Perinatal
1. Parental age at conception	7. Labor/parturition
- low *(mothers < 25 years, fathers < 30 years)*	*prolonged parturition (≥24 h), caesarian section, forceps extraction, vacuum extraction, breech presentation*
- high *(mothers ≥ 35 years, father ≥ 40 years)*	
2. Miscarriages / bleeding	8. Prematurity *(<37 weeks)*
*miscarriages in history, gestational bleeding*	
3. Maternal diseases	9. Birth weight
*diabetes, (pre-) eclampsia, high blood pressure, severe nausea*	- low birth weight *(<2500 g)*
	- high birth weight *(>4500 g)*
4. Maternal infections	10. Suboptimal condition of child at birth
*virus, severe infections*	*low APGAR score at 5 min (<8), respiratory distress, faeces in amniotic fluid, umbilical cord around neck, physical injury*
5. Maternal intoxications	11. Family size/Firstborn^a^
*- alcohol use during pregnancy*	
*- tobacco use during pregnancy*	
6. Stress during pregnancy	12. Pregnancy after fertility treatment
*severe tensions, concerns about the child*	

*Note*. the items underlying the factors are presented in *italic*. For each item, a dichotomous variable was created, coding ‘1’ if the complication was present and ‘0’ if the complication was not present. Then, items were grouped into the pre/perinatal factors based on related content. If at least one complication was present during pregnancy or delivery, ‘1’ was coded on the overlapping factor

^a^Because siblings were included in this study, we compared mean family size between disorders and examined firstborn-ship in the post hoc analyses only

### Procedure

Questionnaires were filled in separately for each child at home, mostly by the mother of the child. Additional data collection included demographic information, blood samples of all family members and neuropsychological data of the children. The study was approved by the local medical ethics board, and parents and children (12 years and older) signed for informed consent.

### Data Analyses

In the BOA (ASD) study, the pre-and perinatal risk factor questionnaire was part of the standard test protocol and administered to all participating families. Missing data for the ASD study was less than 5 % and was missing at random, Little’s (1988) MCAR test, *χ*2 (69) = 59.40, *p* = 0.789. The full pre-and perinatal risk factor questionnaire was only administered to about 50 % of the participating ADHD families (IMAGE study), since it was added to the test protocol at a later stage. A shorter version of the questionnaire (which did not include the PFFs miscarriages/bleeding, maternal diseases, maternal infection, labor/parturition, stress during pregnancy, suboptimal condition at childbirth, and pregnancy after fertility treatment,) was later sent to the remaining participating families. In the IMAGE cohort, data was not missing completely at random, Little’s (1988) MCAR test, *χ*2 (144) = 176.76, *p* = 0.033. A series of ANOVAs and Chi-Squared tests revealed that children whose families did and did not complete the long version of the questionnaire did not differ on age, sex, estimated TIQ, parent-rated ASD symptoms, teacher-rated ADHD symptoms, and proportion SPX vs. MPX, *p*-values > 0.10. Children who filled in the short version of the PPF questionnaire had slightly lower parent-rated ADHD symptoms (*M* = 57.4, SD = 15.2), than children who did complete the long version (*M* = 60.4, SD = 15.9), *F* (1, 742) = 6.19, *p* = 0.009. However, this is likely explained by the larger proportion of control families within this short version group (40 %), compared to the long version group (31 %), *χ*^2^ (1, *N* = 743) = 5.95, *p* = 0.015. Missing data were not imputed to prevent spurious associations.

First, to examine which PPFs were associated with ASD, ADHD or both, Wald chi-square values were calculated using generalized estimated equations (GEE) with a binary logistic model, robust estimators, and exchangeable structure for working correlation matrices. To correct for familial dependency within the data set, family number was used a repeated measure. Independent variables were type of disorder (ASD vs. ADHD vs. control) and sex and the two-way interaction type of disorder*sex. The two-way interaction was dropped from the model when non-significant. Sex was added to the model because previous studies reported that pre-/perinatal complications are more prevalent in boys (Lukkari et al. 2012) and because groups differed in percentage males (see Table 1). Odds ratios (ORs) and 95 % confidence intervals (CIs) were calculated, to define small (OR > 1.5), medium (OR > 2.5), and large effects (OR > 4.3) (Cohen 1988). Dependent variables were the PPFs described above and separate analyses were run for each of the dependent variables. Because siblings were included in this study and only one child per family can be firstborn, the factor firstborn could not be compared between ASD vs. ADHD vs. controls. Instead, family size was examined between disorders. The factor firstborn was examined in post hoc analyses described below.

Second, for all PPFs significantly associated with either ASD or ADHD or both post hoc analyses were conducted to test a) whether the effect was specific to affected children or shared between affected and unaffected siblings and b) whether the effect was selectively found in either SPX or MPX families. Similar GEE analyses were run with independent variables a) diagnosis (affected vs. unaffected siblings) and sex or b) type of family (SPX vs. MPX families) and sex. For the factor firstborn, mean family size was included as covariate to account for the number of children per family. The post hoc analyses were run separately for ASD and ADHD cohorts. A False Discovery Rate (FDR) correction with a *q*-value setting of 0.05 was applied to control for multiple testing (Benjamini 2010). Post hoc power calculations revealed that our study had sufficient statistical power to medium to large effects, but we were underpowered to detect small effects, particularly for affected vs. unaffected and SPX vs. MPX comparisons. Because of the small numbers of individuals available for some of the exposures and the hypothesis-generating nature of our study, a distinction was made between significance (i.e., findings that remained significant after multiple testing), nominal significance (i.e., findings that did not remain significant after multiple testing), and trend-level significance, *p* < 0.10. The latter two were reported in order to not miss out on possible relevant findings that can be tested in future studies. All analyses were carried out in SPSS version 20.

## Results

### Larger Family Size is a Shared PPF for ASD and ADHD

Table 3 presents the *χ*^2^ tests and ORs for the PPFs for ASD and ADHD families. Multivariate significant and trend-level multivariate effects were found on six PPFs, namely family size, low parental age, tobacco use during pregnancy, stress during pregnancy, maternal diseases and maternal infections, *p*-values < 0.10. Only one of these PPFs was significantly associated with both disorders, namely the factor family size, ASD families vs. controls: *χ*^2^ (1, *N* = 696) = 28.54, *p* < 0.001, OR = 1.64, 95 % CI 1.37–1.97; ADHD families vs. controls *χ*^2^ (1, *N* = 884) = 27.98, *p* < 0.001, OR = 1.41, 95 % CI 1.24–1.61; ASD vs. ADHD families: *p* = 0.084. Case parents had significantly more children than control families and this did not differ between ASD and ADHD.

**Table 3:**
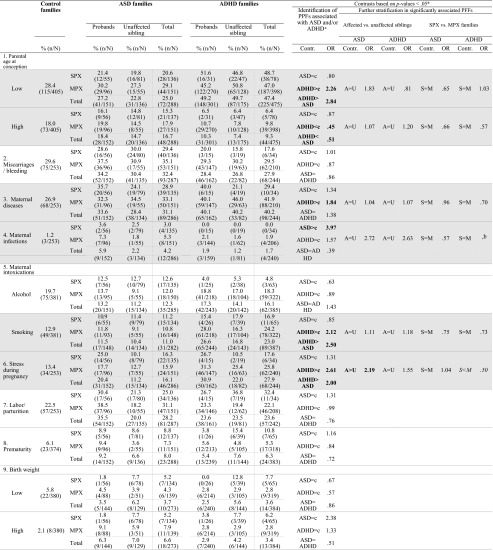

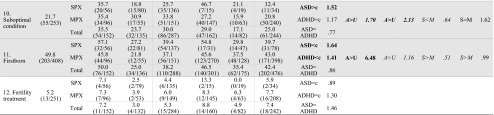
Comparisons between controls on the one hand and individuals from ASD and ADHD families, stratified into affected vs. unaffected siblings and SPX vs. MPX families on the other hand

### PPFs Uniquely Associated With Either ASD or ADHD

Predominantly associated with ASD were the factors maternal infections and suboptimal condition at birth, *χ*^2^ (1, *N* = 539) = 3.83, *p* = 0.050, OR = 3.97, 95 % CI 1.00–15.82, and *χ*^2^ (1, *N* = 540) = 3.88, *p* = 0.049, OR = 1.52, 95 % CI 1.00–2.31, respectively. Children from ASD families were almost four times more likely to have suffered from a severe infection during pregnancy and were one and a half times more likely to have experienced at least one suboptimal condition at birth than controls. These factors were not significantly associated with ADHD. Further, a trend level effect of high birth weight was found in ASD, *χ*^2^(1, *N* = 668) = 2.74, *p* = 0.098, OR = 2.38, 95 % CI 0.85–6.63. Although non-significant, high birth weight appeared to be more frequent in ASD than controls, OR = 2.38.

**Fig. 1:**
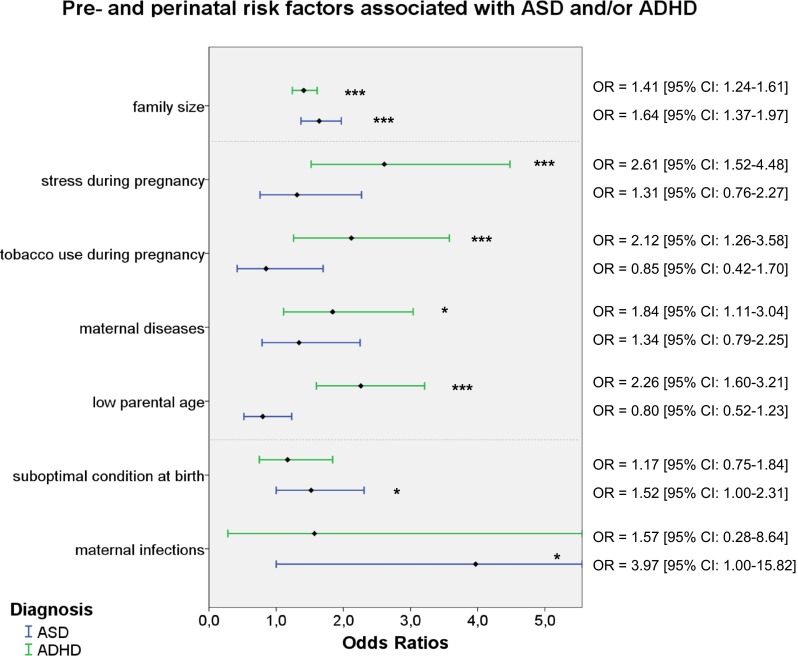
Identified risk factors for individuals from ASD or ADHD families. *Note. ASD* autism spectrum disorders; *ADHD* attention-deficit/hyperactivity disorder. *OR* odds ratio. Represented are the odds that a risk factor was present in children from ASD/ADHD families compared to control children (reference line). Significant odd ratios are indicated with an asterisk (*** *p* < 0.001, * *p* < 0.05)

Predominantly associated with ADHD were the PPFs low parental age, *χ*^2^ (1, *N* = 878) = 21.20, *p* < 0.001, OR = 2.26, 95 % CI 1.60–3.21, tobacco use during pregnancy, *χ*^2^ (1, *N* = 768) = 7.87, *p* = 0.005, OR = 2.12, 95 % CI 1.52–4.48, and stress during pregnancy, *χ*^2^ (1, *N* = 497) = 12.17, *p* < 0.001, OR = 2.61, 95 % CI 1.52–4.48. A nominally significant association was found between ADHD and maternal diseases, *χ*^2^ (1, *N* = 497) = 5.67, *p* = 0.017, OR = 1.84, 95 % CI 1.11–3.04. A reverse effect was found for high parental age, *χ*^2^ (2, *N* = 1168) = 11.51, *p* = 0.003, with parents of ADHD cases being less likely to have an advanced parental age than control parents, *χ*^2^(1, *N* = 878) = 10.85 *p* = 0.001, OR = 0.45, 95 % CI 0.28–0.72. These results indicate that children from ADHD families were over two times more likely to have young parents than control children. Furthermore, case mothers were almost twice as likely to have suffered from at least one disease and were over twice as likely to have smoked or experienced stress during pregnancy as control mothers, see Table 3 and Fig. 1. These factors were not significantly associated with ASD. Moreover, the proportion of children from ADHD families exposed to these four risk factors was significantly higher than that of individuals from ASD families, *p*-values < 0.006.

### PPFs Not Associated With ASD or ADHD

No differences in occurrence between ASD and ADHD cases and controls were found for miscarriages/bleeding, alcohol use during pregnancy, labor/parturition, prematurity, birth weight, and fertility treatment, all *p*-values < 0.10.

### Comparing Significant PPFs in Affected and Unaffected Siblings

**Fig. 2:**
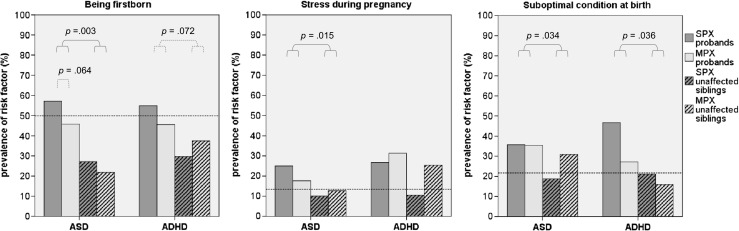
Comparisons between ASD and ADHD probands and their unaffected siblings, stratified for SPX and MPX families. *Note. ASD* autism spectrum disorders; *ADHD* attention-deficit/hyperactivity disorder; *SPX* simplex; *MPX* multiplex. The interpolation lines represent the percentage of control children with the risk factor present. *Dotted lines* indicate trend-level significant findings

As a next step, we compared affected vs. unaffected siblings for their exposure to PPFs. We found that after controlling for family size, ASD affected children were more likely to be firstborn than their unaffected siblings, *χ*^2^ (1, *N* = 288) = 8.77, *p* = 0.003, OR = 6.48, 95 % CI 1.88–22.33. A trend-level effect of being firstborn was found for ADHD as well, *χ*^2^ (1, *N* = 476) = 3.23, *p* = 0.072, OR = 1.16, 95 % CI 0.99–1.35. A suboptimal condition at birth was nominally significantly more frequently reported in ASD-affected compared to ASD-unaffected siblings, *χ*^2^ (1, *N* = 287) = 4.47, *p* = 0.034, OR = 1.70, 95 % CI 1.04–2.79. ASD affected and unaffected offspring did not significantly differ from each other with regard to the prevalence of maternal infections, *p* = 0.114. Within ADHD families, no significant differences were found between ADHD-affected and unaffected siblings for any of the identified PPFs, *p*-values > 0.10. However, despite suboptimal condition at birth not being significantly associated with ADHD, this perinatal complication was nominally significantly more often reported in ADHD-affected compared to ADHD-unaffected offspring, *χ*^2^ (1, *N* = 244) = 4.39, *p* = 0.036, OR = 2.13, 95 % CI 1.05–4.33. Similarly, stress during pregnancy was more frequent in ASD-affected vs. ASD-unaffected children, *χ*^2^ (1, *N* = 286) = 5.91, *p* = 0.015, OR = 2.19, 95 % CI 1.16–4.13, see Fig. 2.

### Comparing Significant PPFs in SPX Versus MPX Families

A final step in the data analyses was the stratification into SPX vs. MPX families. This revealed a trend-level significant difference between SPX and MPX ASD families in the factor being firstborn, *χ*^2^ (1, *N* = 288) = 3.44, *p* = 0.064, OR = 0.51, 95 % CI 0.24–1.04, with SPX ASD families having proportionally more firstborns than MPX ASD families. Further, trend-level effects were found for SPX versus MPX ASD unaffected siblings on suboptimal condition, *χ*^2^ (1, *N* = 135) = 5.91, *p* = 0.052, OR = 0.44, 95 % CI 0.19–1.01, and for SPX versus MPX ADHD siblings on being firstborn, *χ*^2^ (1, *N* = 175) = 3.35, *p* = 0.067, OR = 0.51, 95 % CI 0.24–1.05. Siblings from MPX families were more likely to be firstborn (ADHD) or to have experienced a suboptimal condition at birth (ASD) than unaffected siblings from SPX families, see Fig. 2.

## Discussion

This is the first study to test sharing and uniqueness of the involvement of PPFs for ASD and ADHD, using an approach that stratifies the sample into affected versus unaffected offspring and SPX versus MPX affected families. In accordance with other studies, our findings indicate that pre- and perinatal complications are more frequent in children with ASD and ADHD compared to control children. Novel findings are that we showed that, except for large family size and more firstborns amongst affected offspring, no shared PFFs were identified for ASD and ADHD. PPFs predominantly related to ASD (maternal infections and suboptimal condition at birth) were more often reported in affected offspring. PPFs associated with ADHD (low parental age, maternal diseases, maternal smoking and maternal stress) were shared between affected and unaffected siblings. Furthermore, by using the SPX-MPX stratification, we showed that PPFs in ADHD appear to index an increased shared familial risk, whereas in ASD PPFs possibly have a more determining role in the disorder.

This study adds importantly to the literature by demonstrating that there are limited shared pre- and perinatal risk factors across ADHD and ASD. This is surprising given that cross-disorder traits were present in both ASD and ADHD affected and unaffected siblings, mainly in MPX families (see sample characteristics). This may suggest that the comorbid presence of ASD and ADHD symptoms is not likely explained by shared pre-and perinatal risk factors. The finding that affected siblings were more often firstborns than unaffected siblings concurs with previous literature (Marin et al. 2012; Gardener et al. 2009). This suggests that being firstborn may increase the risk for ASD and ADHD alike, and possibly increase the risk for psychopathology in general (Feehan et al. 1994). Other alternative (or additional) explanations for the increased risk of developing ASD or ADHD as a firstborn have also been suggested. For instance, becoming a parent for the first time is life-changing and puts high demands on parents. Parents are often more anxious and restrictive with the first child than with later children (Eisenman 1992) and firstborn children (particularly boys) experience significantly higher ineffective parenting behaviors as compared to children without siblings or those with older siblings (Arim et al. 2012). Interestingly, family size was larger in families including children with ASD or ADHD. Large family size has been previously associated with ADHD (Biederman et al. 1995) and has been linked to negative child outcomes such as lower average educational levels (Booth and Hiau 2009). It is unclear why case families appear to have more children than control families. These differences in family size may reflect differences in social economic status, religious beliefs, or impulsivity, but this was not tested in this study.

Several PPFs were either predominantly associated with ASD (maternal infections and suboptimal condition at birth) or ADHD (low parental age, maternal diseases, smoking during pregnancy, and stress during pregnancy). Higher frequencies of maternal infections and suboptimal conditions at birth in ASD cases are consistent with previous findings (Gardener et al. 2011; Visser et al. 2013). These factors are likely to reflect immune dysfunction and hypoxia, impacting on neurodevelopment (Onore et al. 2012). Young maternal age is a known risk factor for behavioral problems and has been previously linked to ADHD (Gustafsson and Kallen 2011). The finding that maternal smoking and stress during pregnancy were significantly associated with ADHD also corroborates with previous findings (Thapar et al. 2013). Some studies have reported GxE interactions between some key ADHD risk genes (DAT1, DRD4), maternal smoking (Neuman et al. 2007), and maternal stress (Grizenko et al. 2012), others did not (Altink et al. 2009). Whether maternal smoking and stress are causal agents or proxy variables for the genetic risk to develop ADHD remains unclear (D’Onofrio et al. 2013). The link between maternal tobacco use or maternal stress and offspring ADHD might be attributable to transmission of ADHD risk genes, in addition to any true environmentally mediated effect (Thapar et al. 2013). Proposed mechanism linking stress during pregnancy and ADHD include a disruption in stress-response systems and prefrontal cortex development (Class et al. 2014). Last, maternal diseases such as gestational diabetes mellitus (GDM) and preeclampsia have been previously associated with ADHD (Nomura et al. 2012; Ornoy 2005). Maternal diseases might impact fetal brain growth, possibly resulting in greater inattention and hyperactivity (Ornoy 2005). Our study adds importantly to the existing literature by showing that PPFs are relatively specific for ASD and ADHD. These findings might suggest that the strong overlap between the disorders is unlikely to be caused by many overlapping pre- or perinatal risk factors.

More differentiation in the role of PPFs contributing to ASD and ADHD was found when stratifying the sample into affected versus unaffected children. In ADHD, all PPFs were shared between affected and unaffected siblings. This corroborates previous findings that some PPFs in ADHD (i.e., maternal smoking and alcohol use during pregnancy) did not differ among affected and unaffected siblings (Ben Amor et al. 2005). This was not the case in ASD. Here, PPFs were more prevalent in autistic children compared to their unaffected siblings (Deykin and MacMahon 1980). This suggests that PPFs in ASD may have a unique, highly penetrant contribution to the disorder and are more likely to be true determinants (i.e., state factors), whereas in the case of ADHD, PPFs are weak risk factors that only slightly increase the overall liability for the disorder in a family (trait factors).

Further stratification into SPX and MPX families had some additional value in understanding the role of PPFs, especially for ASD. SPX and MPX ASD families differed with respect to birth order and suboptimal conditions at birth. In SPX ASD, complications during childbirth were more frequent in affected children only, suggesting that these factors may explain why that child did develop ASD, yet his/her siblings did not. In MPX ASD however, a heightened incidence of suboptimal birth conditions was shared between affected and unaffected offspring. This may point to potential pre-perinatal etiological differences between SPX and MPX forms of ASD. Differences in birth rank effects between SPX and MPX ASD families were previously examined, but the results were opposite to ours (Turner et al. 2011), with middle children (particularly those born second) having a higher risk of developing autism than other children in MPX families and increasing risk with each additional birth in SPX families. It was argued that the latter finding might be explained by a higher number of (possibly causative) de novo mutations due to increasing parental age. It is a challenge to explain our finding that SPX affected offspring was more often firstborn than unaffected offspring. Possibly, gene x environment interactions and purely environmental mechanisms for the development of ASD are different for the two types of ASD families. SPX and MPX ADHD could not be significantly dissociated from each other regarding PPFs. These results suggest that SPX-MPX stratification is more suitable to differentiate effects of PPF in ASD families, but in its current form provided little further insight in the role of PPFs in ADHD. However, the limited sample sizes of SPX ADHD families in combination with the low exposure rates for some of the PPFs resulted in a lack of power to detect effects of small size. Based on these non-significant but small effects (OR > 1.5), some risk factors appear to be more frequently shared by affected and unaffected siblings from MPX than SPX ADHD, e.g., maternal diseases, stress during pregnancy and suboptimal birth conditions. Surely, more research is needed to confirm this hypothesis.

Of note, no evidence was found for the association of low birth weight with ASD or ADHD, which was the most promising common risk factor based on previous reports (Ben Amor et al. 2005; Gardener et al. 2011). A possible explanation might be that low birth weight is often associated with advanced maternal and paternal age (Shah et al. 2010), and the proportion of mothers older than 35 years at conception was significantly smaller in case mothers than in control mothers in our study. We could not replicate the consistently reported finding of advanced parental age being associated with ASD in our sample (Gardener et al. 2009). Also, no support was found for maternal smoking being a shared PPF for ASD and ADHD. Previous studies reported that maternal smoking might be specifically related to PDD-NOS, but not (childhood) autistic disorder or Asperger’s syndrome (Visser et al. 2013). In our sample, we did not examine subgroups within ASD, which might have diluted the effect. Alternatively, among the ASDs, PDD-NOS is the most difficult subtype to distinguish from ADHD, and maternal smoking may actually be a unique (genetically driven) PPF for ADHD (Jaspers et al. 2013).

A number of limitations of this study should be considered when weighing the results. First, this study should be viewed as a first step towards a better understanding of the unique and shared contribution of PPFs to the development of sporadic or common ASD and/or ADHD. However, the combination of limited sample sizes of SPX ADHD families in combination with the low exposure rates and interdependence for some of the PPFs resulted in decreased power to detect some true group differences. Therefore, our results should be interpreted with caution. A large majority of ADHD cases stem from MPX families, SPX ADHD families seem to be a relatively rare phenomenon in itself (15.3 % in our sample). Strategic oversampling of SPX ADHD families might be necessary to increase the power of studies using the SPX-MPX stratification. A second important limitation is that our study design was not suited to test causal inference, thus caution is required in assuming that PPFs have causal effects on ASD and ADHD. As Thapar and Rutter (2009) pointed out previously, significant associations between pre/perinatal risk factors and psychiatric disorders may arise because of postnatal risks (e.g., parent mental health problems, social adversity) or through unmeasured confounders including maternally transmitted inherited factors (Thapar and Rutter 2009). Therefore, additional studies indeed using natural experiments such as in Rice et al. (2010) and Thapar et al. (2009), even though highly difficult to undertake, are needed to further test whether the identified PFFs are true causal factors for ASD and ADHD. We feel that our study nonetheless adds to the current literature because to our knowledge, this is the first study that examined (a) overlapping PFFs for ASD and ADHD, and (b) whether risk factors are shared or non-shared between affected and unaffected siblings which may lead to important conclusions about the role of a risk factor in the development of a disorder. Third, the pre-/perinatal data was collected retrospectively which could have resulted in a recall bias when comparing cases to controls. However, previous research shows a reasonable agreement between parent report and medical records for most birth-related data (Buka et al. 2004), and it is unlikely to explain our main finding of PPFs being rather specific for ASD and ADHD. Fourth, because a shorter form of the questionnaire was administered in about half of the ADHD families, small numbers of individuals were available for some of the exposures. However, we believe that insofar this has affected our results, it would likely lead to underestimation of the effect of pre-and perinatal complications on ADHD since we identified significant associations between ADHD and four PPFs. Fifth, the comorbidity of ASD in ADHD families and vice versa, ADHD in ASD families was high. We believe that insofar that this would have affected our results, this would have biased the results towards finding many shared PPFs and not towards finding no or limited shared PPFs, which was not what we found. Therefore, we believe that we can maintain our statement that the high co-morbidity is not likely to be explained by shared PPFs. Last, boys were overrepresented in the clinical samples compared to the control cohort and previous studies report that pre/perinatal complications are more prevalent in boys (Lukkari et al. 2012). Therefore we controlled for sex in our analyses.

In conclusion, the findings reported here indicate that pre- and perinatal complications are more frequent in children with ASD and ADHD compared to control children. Most of the PPFs were uniquely related to either ASD or ADHD, suggesting that the high co-morbidity is not likely to be explained by shared pre-and perinatal risk factors. Instead, PPFs might play a crucial role in the developmental pathways discriminating the disorders on a background of shared genetic factors. Further stratification into affected versus unaffected siblings suggests that PPFs in ASD may have a unique, highly penetrant contribution to the disorder and are more likely to be true determinants (i.e., state factors), whereas in the case of ADHD, PPFs are weak risk factors that only slightly increase the overall liability for the disorder in a family (trait factors). In agreement with our hypothesis, some PPFs appeared to be more frequently shared by affected and unaffected siblings from MPX than SPX families, particularly in ASD. This may pinpoint to potential pre-perinatal etiological differences between SPX and MPX forms of the disorder. These results can stimulate further research on the complex etiologies of ASD and ADHD and the role of PPFs herein.

## Electronic supplementary material

Below is the link to the electronic supplementary material.

ESM 1 (PDF 152 kb)
