# Supplementary material for: Identifying Unique Versus Shared Pre- and Perinatal Risk Factors for ASD and ADHD Using a Simplex-Multiplex Stratification
Source: J Abnorm Child Psychol. 2015 Oct 14;44:923–35. doi: 10.1007/s10802-015-0081-0 (PMC4893356; doi:10.1007/s10802-015-0081-0)
Supplement: Supplementary file 1 — (PDF 152 kb) [file 10802_2015_81_MOESM1_ESM.pdf]

Journal of Abnormal Child Psychology

Identifying unique versus shared pre- and perinatal risk factors for ASD and ADHD using a simplex-multiplex stratification

Anoek M. Oerlemans, Marlot J. Burmanje, Barbara Franke, Jan K. Buitelaar, Catharina A. Hartman, Nanda N.J. Rommelse

Contact information for corresponding author:

Anoek M. Oerlemans, Department of Cognitive Neuroscience, Donders Institute for Brain, Cognition and Behavior, Radboud university medical center, Nijmegen, The Netherlands; and Karakter Child and Adolescent Psychiatry University Centre, Reinier Postlaan 12, 6525 GC Nijmegen, the Netherlands, Tel: +31 24-351 2222, Fax: +31 24-351 2211, Email: Anoek.Sluiter-Oerlemans@radboudumc.nl

SUPPLEMENTAL TABLE 1. Details on child and parent phenotyping and family classification

Child	The exact screening procedures and measures for ASD and ADHD phenotyping in children have been described in previous publications which can be consulted for greater detail (van Steijn et al., 2012). The parent and teacher Social Communication Questionnaire (SCQ) (Rutter et al., 2003), the Child Social Behavior Questionnaire (CSBQ) (Hartman, Luteijn, Serra, & Minderaa, 2006), and Conners Rating Scales Revised (CPRS; CTRS) (Conners, 1996) were used to identify children with ASD and/or ADHD symptoms. These questionnaires are validated instruments to measure ASD and ADHD traits (Charman et al., 2007; Conners, Sitarenios, Parker, & Epstein, 1998; Rutter et al., 2003). All children scoring above cut-off on any of the questionnaires underwent full diagnostic ASD and ADHD assessment, including the Autism Diagnostic Interview-Revised (ADI-R) (Le Couteur, Lord, & Rutter, 2003) and Parental Account of Childhood Symptoms ADHD subversion (PACS) (Taylor, Sandberg, Thorley, & Giles, 1991). Control children were required to obtain non-clinical scores in order to be accepted in the study.
Parent	In the ASD cohort, case and control parents were screened for ASD using the Autism Spectrum Quotient (AQ) (Baron-Cohen, Wheelwright, Skinner, Martin, & Clubley, 2001) and the Adult Social Behavior Questionnaire (ASBQ) (Horwitz, Systema, Ketelaars, & Wiersma, 2005). The ASBQ is the adult version of the CSBQ and, although still under development, shows first promising results in terms of reliability and validity for the ASBQ (Horwitz et al., submitted). In the ADHD cohort, case and control parents were screened for ADHD using the self-report questionnaire for ADHD (Kooij et al., 2005), the self and spouse Conners Adult Rating Scales Self Report (CAARS:S-L) (Conners, Erhardt, & Sparrow,

	1998, 1999), and the Schedule for Affective Disorders and Schizophrenia for School-Age Children - Present and Lifetime Version (K-SADS-PL; administered in follow-up study [NeuroIMAGE]) (Kaufman et al., 1997). Parents scoring above cut-off (Hoekstra, Bartels, Cath, & Boomsma, 2008; Kooij et al., 2005) on any of the ASD/ADHD questionnaires, or on the semi-structured ADHD interview, were considered 'affected'**.** Control parents were required to obtain non-clinical scores in order to be accepted in the study.
Family classification	Families were then stratified into SPX and MPX families, see Oerlemans, Hartman, de Bruijn, Van Steijn et al., 2015, Figure 1. SPX families were required to have a single-affected proband, a minimum of one male sibling and all siblings and parents of the proband unaffected by ASD or ADHD on the basis of non-clinical scores on the screenings questionnaires and/or administered diagnostic interviews. Families with siblings and/or parents who displayed (sub) threshold ASD and/or ADHD symptoms, in addition to the proband, were categorized as multiplex (MPX). Families were excluded if a) only one unaffected parent from a presumed SPX family based on number of affected children participated in this study (to minimize the risk of erroneous categorization because of missing parental data) and b) if the affected proband had only female unaffected siblings (to account for higher sibling recurrence risk in male siblings than female siblings).

Note. ASD = autism spectrum disorders; ADHD = attention-deficit/hyperactivity disorder; SPX = simplex; MPX = multiplex

References

Baron-Cohen, S., Wheelwright, S., Skinner, R., Martin, J., & Clubley, E. (2001). The autism-spectrum quotient (AQ): evidence from Asperger syndrome/high-functioning autism, males and females, scientists and mathematicians. *Journal of Autism and Developmental Disorders, 31*, 5-17.

Charman, T., Baird, G., Simonoff, E., Loucas, T., Chandler, S., Meldrum, D., & Pickles, A. (2007). Efficacy of three screening instruments in the identification of autistic-spectrum disorders. *British Journal of Psychiatry, 191*, 554-559. doi: 10.1192/bjp.bp.107.040196

Conners, C. K., Erhardt, D., & Sparrow, E. (1998). *Conners' adult rating scales-self-report: Long version (CAARS-S:L)*. North Tonawanda, New York: Multi-health systems.

Conners, C. K., Erhardt, D., & Sparrow, E. (1999). *Conners' adult ADHD rating scales technical manual*. New York: Multi-health Systems.

Conners, C. K., Sitarenios, G., Parker, J. D., & Epstein, J. N. (1998). The revised Conners' Parent Rating Scale (CPRS-R): factor structure, reliability, and criterion validity. *Journal of Abnormal Child Psychology, 26*, 257-268.

Conners, C. K. (1996). *Rating scales in ADHD*. Durham, North Carolina: Duke University Medical Center.

Hartman, C. A., Luteijn, E., Serra, M., & Minderaa, R. (2006). Refinement of the Children's Social Behavior Questionnaire (CSBQ): an instrument that describes the diverse problems seen in milder forms of PDD. *Journal of Autism and Developmental Disorders, 36*, 325-342. doi: 10.1007/s10803-005-0072-z

Hoekstra, R. A., Bartels, M., Cath, D. C., & Boomsma, D. I. (2008). Factor structure, reliability and criterion validity of the Autism-Spectrum Quotient (AQ): a study in Dutch population and patient groups. *Journal of Autism and Developmental Disorders, 38*, 1555-1566. doi: 10.1007/s10803-008-0538-x

Horwitz, E. H., Schoevers, R. A., Ketelaars, C. E. J., Kan, C. C., Van Lammeren, A. M. D. N., Meesters, Y., … Hartman, C.A. (submitted). Clinical assessment of ASD in adults using self- and other-report: psychometric properties and validity of the Adult Social Behavior Questionnaire (ASBQ).

Horwitz, E. H., Systema, S., Ketelaars, C. E. J., & Wiersma, D. (2005). Twee zelfrapportagescreeningsvragenlijsten voor autismespectrumstoornissen bij volwassen. *Tijdschrift voor Psychiatrie, 47*, 291-298.

Kaufman, J., Birmaher, B., Brent, D., Rao, U., Flynn, C., Moreci, P., . . . Ryan, N. (1997). Schedule for Affective Disorders and Schizophrenia for School-Age Children-Present and Lifetime Version (K-SADS-PL): initial reliability and validity data. *Journal of the American Academy of Child and Adolescent Psychiatry, 36,* 980-988. doi: 10.1097/00004583-199707000-00021

Kooij, J. J., Buitelaar, J. K., van den Oord, E. J., Furer, J. W., Rijnders, C. A., & Hodiamont, P. P. (2005). Internal and external validity of attention-deficit hyperactivity disorder in a population-based sample of adults. *Psychological Medicine, 35,* 817-827.

Le Couteur, A., Lord, C., & Rutter, M. (2003). *The Autism Diagnostic Interview—Revised (ADI-R).* Los Angeles: CA: Western Psychological Services.

Oerlemans, A. M., Hartman, C. A., De Bruijn, Y. G., Van Steijn, D. J., Franke, B., Buitelaar, J. K., & Rommelse, N.N. (2015). Simplex and multiplex stratification in ASD and ADHD families: a promising approach for identifying overlapping and unique underpinnings of ASD and ADHD? *Journal of Autism and Developmental Disorders, 45,* 645-657. doi:10.1007/s10803-014-2220-9

Rutter, M., Bailey, A., Berument, S., Lecouter, A., Lord, C., & Pickles, A. (2003). *Social Communication Questionnaire (SCQ)*. Los Angeles, CA: Western Psychological Services.

Taylor, E., Sandberg, S., Thorley, G., & Giles, S. (1991). *The epidemiology of childhood hyperactivity*. New York: Oxford University.

van Steijn, D. J., Richards, J. S., Oerlemans, A. M., de Ruiter, S. W., van Aken, M. A., Franke, B., . . . Rommelse, N. N. (2012). The co-occurrence of autism spectrum disorder and attention-deficit/hyperactivity disorder symptoms in parents of children with ASD or ASD with ADHD. *Journal of Child Psychology and Psychiatry and Allied Disciplines, 53*, 954-963. doi: 10.1111/j.1469-7610.2012.02556.x
